# Interpreting GC content differences across populations at polymorphic sites

**DOI:** 10.64898/2026.05.16.725686

**Published:** 2026-05-18

**Authors:** Sheel Chandra, Ziyue Gao

**Affiliations:** 1 Department of Biology, University of Pennsylvania, Philadelphia, PA, USA, 19104; 2 Department of Genetics, University of Pennsylvania Perelman School of Medicine, Philadelphia, PA, USA, 19104

## Abstract

Recent studies have reported consistent inter-population differences in GC content at polymorphic sites in multiple species, including humans. Specifically, populations that experienced recent bottlenecks exhibit lower average GC content (GC%) at common polymorphic sites compared to non-bottlenecked groups—an observation previously interpreted as indication of rapid evolution of base composition. In this study, we investigate the evolutionary and technical factors driving these patterns across humans, mice, maize, and silkworm. We find that GC% at polymorphic sites is highly sensitive to the allele frequency threshold applied. Relaxing this threshold reduces inter-population differences to negligible levels in humans and significantly attenuates similar signals in other species. We further observe substantial GC% variation across allele frequency bins, a pattern driven by the differential abundance of different mutation types. We demonstrate that these observations are collectively driven by an interaction between demographic history and a universal excess of strong-to-weak mutations relative to weak-to-strong mutations, which is counteracted by GC-biased gene conversion (gBGC) over long evolutionary timescales. Forward-in-time simulations with realistic parameters recapitulate observed patterns of GC% variation across both populations and allele frequency bins. Overall, our findings reveal that the base composition at polymorphic sites is strongly shaped by the interaction between demographic history, mutation bias, and gBGC, and does not represent stable, genome-wide trends. Consequently, inter-population differences in GC content—especially at common variants—should not be interpreted as evidence of ongoing divergence in base composition or shifts in mutation patterns.

## Introduction

DNA base composition, a fundamental attribute of the genome, can vary across species but can also be surprisingly conserved between some distantly related species (Bernardi, 2000; Šmarda et al., 2014; Long et al., 2018). Understanding the evolutionary basis of these patterns can help shed light on several related genomic features, such as codon usage, methylation, and genome organization (Costantini et al., 2009; Tillo & Hughes, 2009; Plotkin & Kudla, 2011). The evolutionary forces governing these patterns have been a subject of heated debate, centered on whether genomic GC content (GC%) variation is primarily shaped by neutral mutational processes or by non-neutral forces such as natural selection.

Conceptually, the GC content of a genome is shaped by a balance of conversions between strong nucleotides (C or G) and weak nucleotides (A or T) across generations. Specifically, this is determined by both the mutation rates (μ) and the fixation probabilities (ρ) of strong-to-weak (S>W) and weak-to-strong (W>S) substitutions. For a genome at GC% equilibrium, the number of S>W substitutions should balance the number of W>S substitutions:

$$GC\%\cdot \mu_{S>W}\cdot \rho_{S>W}=AT\%\cdot \mu_{W>S}\cdot \rho_{W>S}$$

When the left-hand side of the equation is greater, more S>W substitutions will be fixed into the genome, reducing the overall GC content and hence the value of the left-hand side. Conversely, when the right-hand side is greater, the genomic GC content will increase until the balance is achieved.

Single nucleotide mutation rates strongly depend on the mutation type and sequence context (Hwang & Green, 2004; Lynch, 2010). Typically, the average rate of S>W mutations is higher than that of W>S mutations (Lynch, 2010). This S>W mutation bias can be partially attributed to the hypermutability of methylated cytosines but is also observed in species lacking DNA methylation (Petrov & Hartl, 1999).

Intriguingly, even in terms of mutation counts, many species exhibit a consistent excess of S>W over W>S mutations, which has been observed in natural polymorphisms, *de novo* mutations, or mutation accumulation experiments (Keightley et al., 2009; Hershberg & Petrov, 2010; Bergeron et al., 2021). This universal imbalance suggests that mutational input alone would drive genomes toward a more AT-rich state, implying either that these genomes are far from GC equilibrium or that a compensatory fixation bias ($\rho_{W>S}>\rho_{S>W}$) counteracts the mutational pressure.

Indeed, the probability of fixation for S>W and W>S alleles can differ due to differences in the direction, strength, and rate of natural selection as well as biased gene conversion. In genomes with large fractions of non-coding regions, differences in average fitness effects between S>W and W>S variants are likely negligible. However, GC-biased gene conversion (gBGC) is widespread across diverse taxa, including yeasts, plants, fish, birds and mammals, significantly influencing genetic variation patterns and GC content evolution (Mancera et al., 2008; Duret & Galtier, 2009; Muyle et al., 2011; Nabholz et al., 2011; Figuet et al., 2015; Glémin et al., 2015; Boman et al., 2021). gBGC occurs due to the biased repair of mismatches in heteroduplex recombination intermediates during meiosis, leading to a slight over-transmission of GC alleles over AT alleles in one generation and a greater fixation probability for W>S variants over evolutionary timescales. Notably, the effects of gBGC on the fixation probabilities of different mutation types depend on recombination rate, transmission bias, and effective population size (Duret & Galtier, 2009).

Recently, a study (Li et al., 2015) reported striking differences in GC content (GC%) at polymorphic sites between populations within the same species. Specifically, individuals in groups that experienced recent bottlenecks and/or domestication (referred to as the “derived groups” in (Li et al., 2015) showed lower GC% at single nucleotide polymorphisms (SNPs) with minor allele frequency (MAF) above 5% compared to those in non-bottlenecked groups (referred to as the “ancestral groups” in the study). This pattern is consistently observed across a wide range of species, including humans, mice, insects, and a variety of plants (Wang et al., 2019; Zhao et al., 2020; Gou et al., 2024), which was interpreted as indication of rapid shifts in GC%, potentially foreshadowing divergence in base composition between populations.

To explain their observed inter-population differences, Li et al. hypothesized that bottlenecked populations may have accumulated mutations in DNA repair genes, resulting in a stronger S>W mutation bias. However, this explanation seems unlikely, at least for humans, for several reasons. First, known mutation-spectrum differences among populations are modest and context-specific, and they are unlikely to substantially alter the overall S>W mutation bias (Harris, 2015; Harris & Pritchard, 2017; Mathieson & Reich, 2017; Gao et al., 2023). Second, the strongest signal of mutation spectrum shifts, a European-specific TCC>TTC mutation pulse, is not found in East Asians or Americans and does not match the African versus non-African differences in GC%. Finally, new mutations that arose after population separation are expected to be rare and population-private, and unlikely to contribute substantially to globally common SNPs (Biddanda et al., 2020). Another possibility briefly mentioned by Li et al. is differential fixation of variants between bottlenecked and non-bottlenecked populations, but no specific evolutionary mechanisms (i.e., drift, gene conversion, or selection) were discussed, nor was how population demographic history could influence (or correlate with) these evolutionary forces.

In this study, we investigated whether the observed inter-population differences in GC content are robust and sought to identify the technical factors or evolutionary forces driving these patterns. Our analysis reveals that inter-population GC% differences at polymorphic sites are highly sensitive to the allele frequency threshold applied—a pattern we consistently observed across human, mouse, maize, and silkworm. When the allele frequency cutoff was relaxed, the inter-population differences in GC content drastically reduced across all species studied. We propose a parsimonious model in which the observed differences emerge from the interaction between a universal excess of S>W mutations, GC-biased gene conversion, and population-specific demographic impacts on the site frequency spectrum. Overall, our findings demonstrate that common variants are strongly shaped by demographic history and are not representative of genome-wide patterns in base composition.

## Results

### GC content differences between human populations depend strongly on allele frequency threshold

We first replicated the observed differences in GC% between human populations using the high-coverage whole genome sequencing in the updated 1000 Genomes Project dataset (Byrska-Bishop et al., 2022). Following the methodology of Li et al., we initially filtered for common variants, defined as SNPs with a global minor allele frequency (MAF) of ≥ 5%, after excluding individuals with clear admixed continental ancestries (population labels ACB, ASW, PUR, CLM, and PEL; see Methods). To minimize the effects of natural selection, we restricted our analysis to putatively neutral regions by excluding conserved and coding sequences. After filtering, the dataset contained 2064 individuals (504 AFR, 503 EUR, 504 EAS, 489 SAS, 64 AMR) and 2,673,086 qualifying common SNPs.

Focusing on these common SNPs, we quantified the base composition on the reference strand for each individual using an allele-counting approach. Specifically, for each qualifying polymorphic site (defined based on the allele frequency in the entire dataset), we counted the contribution of two alleles to the individual’s total base counts based on their genotype value; for instance, a homozygous SNP with two alternative T alleles contributes two to the aggregate T count, while a heterozygous SNP with C and A alleles contributes one C and one A to the aggregate counts. We then calculated the base composition by totaling the counts of A, C, G, and T across all qualifying SNPs for each individual and. Following Li et al., we plotted the fraction of C alleles against the fraction of A alleles. Because this procedure relies solely on observed allele counts rather than relying on ancestral polarization or reference state, the resulting composition estimates are mathematically invariant to the reference genome, ancestral polarization, or population allele frequency.

**Figure 1.**
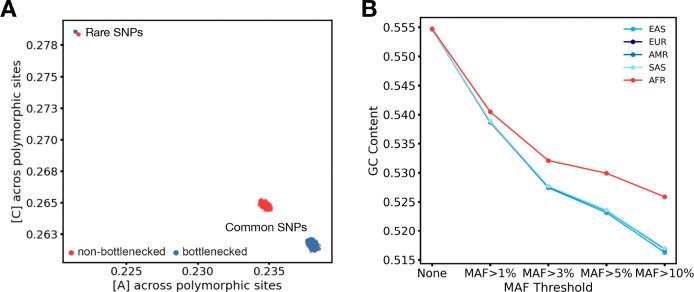
GC% across polymorphic sites is dependent on MAF threshold applied. **A)** Reference strand base composition plotted as proportion of A vs. proportion of C across common polymorphic sites (MAF≥5%) and rare polymorphic sites (MAF<5%) in AFR and non-AFR groups. Individuals with admixed ancestries (i.e., with population labels ‘ACB’, ‘ASW’, ‘PUR’, ‘CLM’, ‘PEL’) are excluded. **B)** GC% across polymorphic sites calculated from different variant sets: No MAF threshold, ≥1%, ≥3%, ≥5%, and ≥10% MAF.

Our analysis replicated the finding of significantly higher GC% in African individuals at common SNPs compared to non-African individuals (Figure 1A, 52.89% vs. 52.27–52.32%; Wilcoxon rank-sum test, p-value < 10^−80^). However, this pattern changed strikingly when rare variants (MAF<5%) were examined: they display substantially higher GC content than common variants. Moreover, the direction of the inter-population difference reversed at rare variants: African individuals have lower GC content than non-African individuals (Figure 1A, 55.65% vs. 55.69%; Wilcoxon rank-sum test, p-value < 10^−80^).

**Figure 2.**
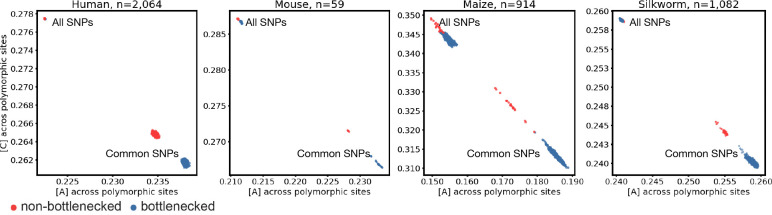
Dramatic decrease in GC% differences between bottlenecked and non-bottlenecked groups when MAF threshold is removed. Reference strand base composition plotted as proportion of A vs. proportion of C across common polymorphic sites (MAF≥5%) and all polymorphic sites (no MAF threshold) between bottlenecked and non-bottlenecked populations in human, mouse, maize, and silkworm. Number of individuals sampled in each polymorphism dataset is indicated in the plot heading.

The opposing trends at common and rare SNPs suggest that the previously reported signal might depend critically on allele frequency conditioning. We therefore quantified the overall GC content across all SNPs. Due to the greater abundance of rare variants, the overall GC% is dominated by patterns of the rare variants for all populations (Table 1). Crucially, the previously reported differences in GC% between African and non-African populations nearly disappeared (Figure 2A, 55.474% for AFR vs 55.466% for non-AFR), leaving only a negligible absolute difference despite statistical detectability in this large dataset.

To further characterize the effect of allele-frequency conditioning, we recalculated GC content across progressively stricter MAF. Inter-population differences emerge only after rare variants are excluded and continue to intensify with increasingly stringent frequency cutoffs, increasing from ~0.009% without MAF filtering to ~1% at MAF ≥10% (Figure 1B). These results demonstrate that common SNPs constitute a biased subset of segregating variants, and GC content differences among human populations are negligible when all segregating variants are considered.

### Including rare variants greatly reduces inter-population GC-content differences across species

To determine whether the frequency-dependence of inter-population GC% differences is a general phenomenon, we extended our analysis to three additional species: mouse (between subspecies *Mus musculus castaneus and M.m.domesticus, M.m.musculus*), maize (between subspecies *Zea mays mays* and *Z.m.parviglumis*), and silkworm (between *Bombyx mori* and *B. mandarina*). These species were previously reported to exhibit reduced GC content in bottlenecked or domesticated populations at common SNPs (Li et al., 2015; Wang et al., 2019; Zhao et al., 2020; Gou et al., 2024). Across all species examined, the magnitude of the inter-population difference depended strongly on the applied allele-frequency threshold (Figure 2, Tables S3–5).

In all four species examined, rare variants consistently exhibited higher GC content than common variants, and crucially, inclusion of rare variants substantially attenuated the previously reported differences between bottlenecked and non-bottlenecked groups (Tables S3–5, Figure 2). This pattern remained qualitatively unchanged when singleton variants were excluded (Figure S1). In human and silkworm, the differences diminished to nearly negligible levels; in mouse and maize, the initial GC% differences observed at MAF ≥5% were reduced in magnitude by ~90% and 76% respectively when all variants were considered. The residual differences in mouse and maize may in part arise from the relatively small sample sizes in these datasets (n = 59 and 914 individuals, respectively), which limit power to capture the rarest segregating variants and may therefore cause common-variant patterns to remain disproportionately represented in aggregate estimates. Nevertheless, the substantial attenuation of inter-population differences after inclusion of rare variants was consistently observed across all species analyzed, indicating that the sensitivity of GC content estimates to allele frequency conditioning is widespread across phylogenetically diverse taxa.

### Demographic history, mutation bias and biased gene conversion jointly shape GC content at SNPs across frequency bins

**Figure 3.**
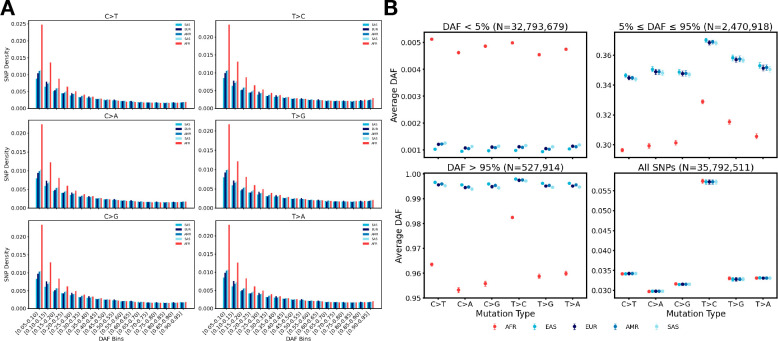
Site frequency spectrum (SFS) and average derived allele frequencies (DAF) for SNPs generated by different mutation types. **A)** SFS for SNPs of each mutation type across five human population groups. Only DAF bins between 5% and 95% are shown to aid visualization. **B)** Average DAFs for each population group across different variant sets stratified by DAF: DAF<5%, 5%≤DAF≤95%, DAF>95%, and all SNPs, for each mutation type. Error bars represent 95% confidence intervals estimated using block bootstrap (see Methods).

The recurrence of the threshold effect across four phylogenetically diverse species suggests that the observed GC-content differences are unlikely to be driven primarily by lineage-specific changes in mutation bias. We therefore examined whether a more general mechanism—interaction between demographic history, mutation bias, and GC-biased gene conversion (gBGC)—could explain the frequency-dependent patterns. We began with human populations, where demographic history is relatively well characterized and genomic data are most comprehensive. To generate the unfolded SFS, we determined the ancestral and derived alleles of each SNP using the inferred ancestral human genome (see Methods) and classified mutations into six types: C>T, C>A, C>G, T>C, T>G, and T>A. As expected under known demographic models, African populations exhibited an enrichment of intermediate-frequency variants (5% < DAF < 45%) and a depletion of low- and high-frequency variants relative to non-African populations across all mutation types (Figure 3A, Figure S2).

To minimize potential artifacts arising from recurrent mutation or mis-inference of ancestral alleles, we repeated this analysis after excluding CpG and TpG sites and obtained qualitatively similar results (Figure S3,4). This indicates that recurrent mutations or ancestral mis-polarization has a minimal impact on the overall patterns observed here.

Because populations differ in their SFS, African and non-African populations also differ systematically in average derived allele frequency (DAF) (Figure 3B, Table 1). For all mutation types, African populations exhibit lower average DAF than do non-AFR populations at common SNPs (i.e., 5≤DAF≤95%) as well as at rare SNPs with DAF>95%, whereas the opposite is true for rare SNPs with DAF<5% (Table S1). At the same time, strong-to-weak (S>W) mutations outnumbered weak-to-strong (W>S) mutations among common and low DAF variants (Table S1). Together, these observations explain the contrasting GC-content patterns across frequency bins: because derived alleles at S>W mutations are disproportionately AT-increasing, bottlenecked populations with higher average DAFs at common variants exhibit lower GC content at those sites (Table 1).

Notably, the average DAFs across all SNPs in aggregation are remarkably similar among populations, despite substantial differences within individual frequency bins (Figure 3B). This cancellation of average DAF differences holds true across all mutation types, irrespective of the mutation types. Consequently, the aggregate GC content across all variants differs only minimally across populations, consistent with our earlier observations (Figure 1). These results indicate that the previously reported GC-content differences at common SNPs primarily arise from demographic distortions of the SFS combined with the universal excess of S>W relative to W>S mutations observed across diverse species (Hershberg & Petrov, 2010; Lynch, 2010; Ossowski et al., 2010).

We also observed similar SFS shifts in mouse and maize, where bottlenecked groups displayed elevated average DAFs at common SNPs (Tables S2 and S3). These cross-species patterns support the same demographic explanation: lower GC content at common SNPs can arise from shifts in the SFS combined with a general excess of S>W over W>S mutations, without requiring population-specific changes in mutation bias.

The same framework also explains why rare variants consistently display higher GC content than common variants across all species analyzed (Figure 1, Tables S1–4). Most rare variants have low copy numbers of the derived alleles, and because S>W mutations substantially outnumber W>S mutations among these variants (Tables S1–S4), their aggregate GC content is dominated by ancestral GC bases. In contrast, derived alleles at common SNPs contribute more substantially to aggregate base composition, reducing the average GC content at these sites.

Why, then, are S>W mutations more abundant than W>S mutations among segregating variants? A natural explanation is gBGC, which leads to fixation bias favoring W>S over S>W alleles. If the genome is already at or very close to equilibrium GC content, S>W and W>S substitutions must be approximately balanced over evolutionary timescales. Because W>S variants have higher fixation probabilities under gBGC, the balance in substitution requires greater mutational input of S>W than W>S variants.

### Simulations with universal mutation bias and gBGC recapitulate observed patterns

To evaluate whether the interaction of demographic history with universal mutation bias and gBGC is sufficient to produce the observed GC% patterns, we performed forward-in-time simulations using SLiM (Haller & Messer, 2023), roughly following an estimated demographic model of human populations (Gravel et al., 2011). The demographic model included three populations representing African, European, and East Asian populations, with a severe out-of-Africa bottleneck in the non-African lineage followed by rapid population expansion.

We implemented a universal mutation bias favoring S>W mutations ($\mu_{S>W}/\mu_{S>S}=2.15$), based on estimates from human *de novo* mutation data (Kong et al., 2012). Because such a mutation bias alone would drive the genome toward a substantially lower equilibrium GC content (~30%), we introduced gBGC into the simulation, with its strength tuned to match the empirical human genome GC% of roughly 41%. Simulations were initiated at this expected equilibrium genomic GC content and ran for 10Na burn-in generations prior to population split to allow the ancestral population to approach equilibrium in both SFS and stable GC% at segregating sites.

**Figure 4.**
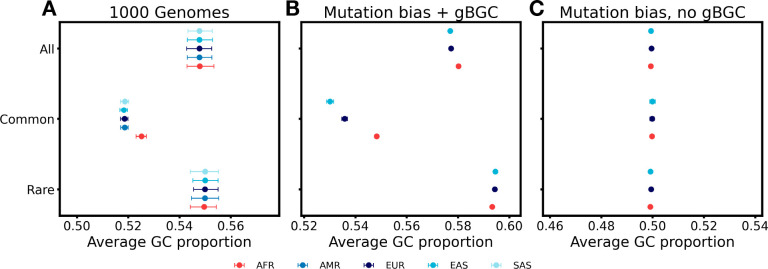
GC% at polymorphic sites from simulating a universal S>W mutation bias and GC-biased gene conversion matches observed patterns from 1000 Genomes across the frequency spectrum. Average GC proportion across polymorphic sites for each population group at common SNPs (MAF>=5%), rare SNPs (MAF<5%), and all SNPs estimated from: **A.** 1000 Genomes; **B.** a simulation scenario with a universal S>W mutation bias $\mu_{S>W}/\mu_{S>S}=\;2.15$ and a GC-biased gene conversion (gBGC) strength tuned to match the empirical human genome GC% of ~41%; and **C.** a simulation scenario with a mutation bias of $\mu_{S>W}/\mu_{S>S}=\;2.15$, no gBGC, and an equilibrium GC% of ~32%. 95% confidence intervals were estimated using block bootstrap.

Our simulation results closely mirror our empirical findings in the 1000 Genomes Project data across the entire frequency spectrum. In general, rare variants exhibited higher GC content than common variants (59.4% vs. 53.8%). The non-bottlenecked African population showed lower average DAF and higher GC% at common SNPs (54.45% for AFR vs. 52.95% and 52.63% for EUR and EAS) but higher DAF and lower GC% at rare SNPs relative to the bottlenecked non-African populations (59.33% for AFR vs. 59.44% and 59.45% for EUR and EAS) (Table 2, Figure 4). Moreover, compared to common SNPs, GC-content differences between populations became substantially attenuated when all variants were considered (58.02% for AFR vs. 57.73%, 57.69% for EUR, EAS), mirroring the empirical observations across human populations.

In contrast, in control simulations incorporating the same mutation bias ($\mu_{W>S}/\mu_{S>W}=2.15$) but excluding gBGC, the genomic GC content converged to the expected mutational equilibrium, producing approximately 50% GC content across all allele frequency bins in all populations (Figure 4). Under this scenario, no systematic GC-content differences emerged between rare and common variants or among populations, indicating that mutation bias alone is insufficient to generate the observed empirical patterns. Together, these results demonstrate that the interaction between demographic history with universal mutation bias and gBGC is sufficient to explain the frequency-dependent GC content patterns observed across populations, without invoking population-specific shifts in mutation spectrum.

## Discussion

### Reinterpreting GC-content differences at polymorphic sites

Previous studies reported lower GC content at common SNPs in bottlenecked or domesticated populations and interpreted this pattern as evidence for rapid shifts in base composition or mutation bias. Our analyses show that this signal is highly dependent on allele-frequency conditioning. Although we replicated the previously reported differences at common variants, the direction reversed among rare variants and nearly disappeared when all variants were considered. This observation aligns with prior analyses of the Simons Genome Diversity Project (SGDP) dataset, which found broadly similar accumulation rates of S>W and W>S mutations across human populations, with the exception of the deeply diverged San population not included in the 1000 Genomes dataset analyzed here (Do et al., 2015). Similar attenuation was observed in mouse, maize, and silkworm, indicating that this effect is not unique to recent human demographic history.

These observations can be explained by a simple population-genetic mechanism. Bottlenecked populations have distorted site frequency spectra, including higher average derived allele frequencies among common SNPs. Because S>W mutations outnumber W>S mutations among segregating variants, this shift lowers GC content at common SNPs in bottlenecked populations. To illustrate this intuition, consider a set of SNPs in which a fraction x are S>W mutations and 1−x are W>S mutations, with both classes having average DAF of f. The average GC content of this SNP set is then GC%=(1−f)+(1−x)f=x+(1−2x)f. Because S>W mutations generally outnumber W>S mutations among segregating variants (x>0.5), GC content decreases as average DAF increases. This same mechanism also explains why rare variants have higher GC content than common variants: rare variants are dominated by low-frequency S>W mutations (i.e., tiny f) whose ancestral alleles are GC bases. Importantly, an excess of S>W mutations among segregating variants does not imply ongoing genome-wide decline in GC content, as this mutational imbalance can be counteracted by gBGC among mutations that eventually fix. Similarly, inter-population differences in the GC% at polymorphic sites, even across all SNPs (Table 2), do not imply divergence in future GC content, as the fixation probabilities of S>W and W>S mutations differ between populations due to demographic influences.

Compared to the “mutation explanation” proposed in Li et al (2015), our “demography explanation” is more parsimonious, as it does not invoke inter-population differences in mutation spectrum or fixation probability. The only assumptions are a general S>W mutation bias and universal gBGC, features observed in most species studied to date (Keightley et al., 2009; Hershberg & Petrov, 2010; Lynch, 2010; Bergeron et al., 2021; Näsvall et al., 2023). Forward simulations combining universal S>W mutation bias, gBGC, and human demographic history recapitulated the observed patterns, providing further support for sufficiency of the “demography explanation”.

### Cross-species variation in the magnitude of the threshold effect

While relaxing allele frequency thresholds reduced inter-population GC% differences across all species, the effect was most pronounced in humans, where differences reached negligible levels. In contrast, maize and mouse exhibited the largest residual signals even when all variants were considered. We hypothesize that the varying attenuation of “inter-population differences” across taxa is driven by the interaction between demographic history (both divergence time and effective population size), recombination landscapes, and technical factors such as sample size.

Biologically, these differences could influence the population-scaled strength of gBGC, which determines the degree to which the site frequency spectra of S>W and W>S variants deviate from neutral expectations. On the other hand, technical constraints such as small sample sizes lack the power to capture the most recent, rare mutations in the population; consequently, the demographic signal at common variants may remain disproportionately represented in the aggregate GC% of all variants, creating apparent differences even in absence of true evolution in mutation patterns.

The evolutionary scale of the population split may also explain the persistence of the residual signal in some species. Unlike the relatively recent human population divergence, the mouse subspecies diverged approximately 350,000 to 500,000 years ago, representing a significantly deeper divergence in terms of drift units (*T/2Ne*) (Geraldes et al., 2011; Fujiwara et al., 2022). In such diverged lineages, a larger fraction of all variants consists of population private mutations that have evolved under independent mutational, demographic and gBGC regimes for millions of generations. Consequently, the “all-SNP” aggregate in the mouse subspecies could reflect genuine biological divergence in base composition accumulated over time, rather than a transient frequency-shifting artifact of a recent bottleneck. However, such hypothesis requires more rigorous testing and systematic exclusion of alternative biological (e.g., differences in recombination rates) or technical explanations (e.g., differences in genome assembly quality).

### Effects of allele frequency filters on GC content at polymorphic sites

The practical implication of our analysis is the substantial influence of the MAF threshold on the quantification of GC content at segregating sites (Figure 1). Rare or ultra-rare variants are commonly excluded from genomic analyses to reduce genotyping error, especially in low-coverage datasets, but our results show that such filtering can strongly bias summaries of base composition at segregating sites. Because frequency-filtered variants are a non-random subset of polymorphisms shaped by demography, mutation bias, and gBGC, their aggregate GC content need not reflect either all segregating variation or genome-wide base composition. As MAF thresholds become more stringent, average GC content declines and subtle inter-population differences can be amplified (Figure 1B).

The flip side of this issue is also important: (ultra-)rare variants, including singletons, are not themselves representative of either all genomic sites or newly arising mutations, because they are conditioned on recent mutation and on observation as polymorphisms in the sample. The first condition enriches segregating sites for more mutable sites and therefore for GC bases, because GC sites are generally more mutable than AT sites ($\mu_{S>N}>\mu_{W>N}$) under typical AT mutation bias ($\mu_{S>W}>\mu_{W>S}$). The second condition means that variants in the same frequency class, such as singletons or doubletons, can differ in allele age across populations due to differences in demographic history and sample size, and therefore are not directly comparable. In general, subtle inter-population differences among rare variants should not be interpreted as evidence for shifts in mutation patterns, unless alternative explanations—including differential gBGC, recurrent mutation, and demographic effects—have been considered and ruled out.

## Materials and Methods

### Data sources and filtering

#### Polymorphism:

We downloaded unphased autosomal polymorphism data from published sources for four species: human (Byrska-Bishop et al., 2022), mice (Harr et al., 2016), silkworm (Tong et al., 2022), and maize (Bukowski et al., 2018). We selected these datasets because (1) they were based on whole-genome sequencing and provided unbiased survey of polymorphisms; (2) the evolutionary relationship of the studied populations is known, allowing for distinction between bottlenecked and non-bottlenecked groups; and (3) adequate numbers of SNPs were detected. For all datasets, we kept only biallelic SNPs. To minimize potential genotyping errors, only SNPs with more than 80% of samples genotyped were considered. Following Li et al., we operationally defined “common variants” as SNPs with minor allele frequency (MAF) greater than or equal to 5% in samples across all populations, “rare variants” as SNPs with MAF less than 5%, and “all variants” as all SNPs with no MAF threshold. To distinguish between bottlenecked and non-bottlenecked groups, we excluded individuals from admixed populations (i.e., with population labels ‘PUR’,’CLM’,’ACB’,’PEL’,’ASW’) in the 1000 Genomes Project in this analysis. We also identified two individuals from inbred maize populations (‘ZEAxppRDLDIAAPEI-2’ and ‘ZEAhwcRAXDIAAPE’) which clustered with wild individuals based on their proportions of A and C bases at polymorphic sites. We hypothesized that these individuals are admixed and excluded them from this analysis.

### Definition of putatively neutral region

To minimize the effects of selection, we defined “putatively neutral regions” in our datasets by excluding coding and conserved regions of the genome when available (see “Resources” section below for sources). Specifically, we excluded coding and phylogenetically conserved regions of the human and mouse genomes. For the maize genome, we only excluded coding regions as annotation of conserved regions was not readily available. We did not apply any filters to the silkworm genome because there is no data for coding or conserved regions to our knowledge. For analysis of human polymorphism data, we further intersected “putatively neutral regions” with the Strict Mask of 1000 Genomes dataset and only focused on SNPs in the intersected regions.

**Table:** 

Species	Dataset	Number of Individuals	Putatively neutral SNPs	Putatively neutral common SNPs
Human	Byrska-Bishop, M. et al. 2022	2,064	35,792,511	2,673,086
Mouse	Harr, B. et al. 2016	59	29,092,618	9,336,832
Maize	Bukowski, R. et al. 2018	914	2,633,942	532,273
Silkworm	Tong, X. et al. 2022	1,082	40,378,020	9,201,012

### Resources

**Table:** 

1000 Genomes Strict Mask	http://ftp.1000genomes.ebi.ac.uk/vol1/ftp/data_collections/1000_genomes_project/working/20160622_genome_mask_GRCh38/StrictMask/
Conserved Regions	Human: https://hgdownload.cse.ucsc.edu/goldenPath/hg38/database/phastConsElements30way.txt.gz Mouse: https://hgdownload.soe.ucsc.edu/goldenPath/mm39/database/phastConsElements35way.txt.gz
Coding Regions	Human: http://hgdownload.soe.ucsc.edu/goldenPath/hg38/database/seqNcbiRefSeq.txt.gz Mouse: https://hgdownload.soe.ucsc.edu/goldenPath/mm39/database/ncbiRefSeq.txt.gz Maize: https://ftp.ncbi.nlm.nih.gov/genomes/all/GCF/000/005/005/GCF_000005005.1_B73_RefGen_v3/GCF_000005005.1_B73_RefGen_v3_feature_table.txt.gz
Ancestral Allele Reconstruction	10 primate alignment (human): https://useast.ensembl.org/info/genome/compara/multiple_genome_alignments.html *est-sfs* (mouse and maize): https://doi.org/10.1534/genetics.118.301120

### Base composition across polymorphic sites

Based on the unphased genotype values in the VCF of 1000 Genomes Project (0/0 for homozygotes for the reference allele, 0/1 or 1/0 for heterozygotes, and 1/1 for homozygotes for the alternative allele), we scored the number of alternative alleles at each site for each individual. We then summed the total numbers of A, C, G, and T bases across common SNPs (MAF>=5%), rare SNPs (MAF<5%), and all SNPs for each individual and computed the proportions of A and C alleles on the reference strand (Figure 1A). We use “Superpopulation” labels assigned to each individual sample in the 1000 Genomes Project to distinguish between five continental ancestry populations: African (‘AFR’), European (‘EUR’), American (‘AMR’), East Asian (‘EAS’), and South Asian (‘SAS’). To assess whether GC content at polymorphic sites differed significantly between bottlenecked and non-bottlenecked individuals, we computed per-individual GC% as (G+C)/(A+T+G+C) for each variant class and applied a Wilcoxon rank-sum test.

To calculate the average GC% for each population in different variant sets (rare, common, or all), we used population-specific AF values estimated from individual genotype information. Further, we used block bootstrap to evaluate the variance in GC% across polymorphic sites. Specifically, we partitioned each SNP set into 100 non-overlapping blocks of roughly equal size, randomly sampled 100 bins with replacement as a replicate, and calculated the average GC% across SNPs for each replicate. We performed the resampling 500 times to get the distribution of GC% for each SNP set and estimated 95% confidence intervals from the 2.5th and 97.5th percentiles of the distribution (Figure 4). To further evaluate the effect of allele frequency thresholding on GC% of SNPs, we applied several MAF filters (1%, 3%, 5%, and 10%) and calculated the GC% in each of the resulting datasets (Figure 1B).

### Ancestral allele polarization

To determine the mutation type that gave rise to each SNP, we used ancestral allele reconstruction based on the 10 primate EPO alignment for humans. For mice and maize, we assigned ancestral states probabilistically using *est-sfs*, which takes SNPs from a focal species (*Mus musculus; Zea Mays*) and an outgroup aligned to the same reference genome (*Mus spretus*; *Tripsacum dactyloides*) to estimate the probability that the major allele of the focal species is the ancestral state (Keightley & Jackson, 2018). For each SNP, the ancestral allele was assigned by a Bernoulli draw weighted by this probability. We then annotated each SNP with the type of substitution: C>T, C>A, C>G, T>C, T>A, and T>G (where each type includes the corresponding reverse complement; e.g., C>T includes both C>T and G>A).

### Site frequency spectrum analysis

For human, mouse, and maize polymorphisms, we determined the DAF for each SNP using the population-specific allele frequencies computed from individual genotype data (see Data sources) and the inferred ancestral allele (see “Ancestral allele polarization”). To further investigate properties of the SFS of each population, we calculated the average DAF across all sites (or across common/rare variants) of each mutation type. Using the population-specific average DAF values, we determined the average AT and GC content in each mutation type based on the identity of the derived and ancestral allele. For example, if the average DAF for all SNPs generated by C>T mutations is X, the average AT content per individual across these sites would be X and GC content (1−X). We used the same bootstrap approach as described earlier to generate a distribution of DAF in each variant set for every mutation type and estimated the 95% confidence intervals as the 2.5th and 97.5th percentiles of the distributions (Figure 3B).

### Excluding CpG and TpG sites

Since CpG sites have more than 10x higher transition mutation rates than the genome average, CpG and TpG sites in present-day populations are more likely to have experienced recurrent mutations, which can affect both polarization of the ancestral alleles and quantification of the SFS (for example, two variants at frequencies 1% and 2% due to independent, recurrent mutations at the same site would be treated as one variant at 3%). To evaluate these effects, we computed SFS for common C/T polymorphisms excluding CpG/TpG sites. We extracted the 5’ and 3’ flanking nucleotides at each SNP from the GRCh38 reference genome and excluded all sites at which either the reference or the alternative allele could form a CpG or TpG. From this filtered dataset, we obtained the SFS by population for different mutation types (Figures S3,S4), which were qualitatively similar to Figure 3 and Figure S2. We also generated a table of the average DAF in each mutation type for every population group along with the average GC% across SNPs in that variant set and found that patterns of inter-population differences in GC% and average DAF were consistent whether CpG/TpG sites were excluded or not (Tables S1,S2).

### Forward-in-time simulations of mutation bias and gBGC

To evaluate whether mutation bias and gBGC, coupled with differences in human demographic history, are sufficient to reproduce the observed inter-population differences in GC% at polymorphic sites, we performed forward-in-time simulations using SLiM 4.0.1 (Haller & Messer, 2023). Each simulation consisted of a 1Mb locus with a per-base per-generation mutation rate of μ=6.89×10^−9^ and a recombination rate of 1×10^−8^ per base per generation. Mutation bias was implemented via a nucleotide mutation rate matrix in which all weak-to-strong (W>S, i.e. G/C>A/T) mutations were assigned a relative rate of 1.0 and all strong-to-weak (S>W, i.e. A/T>G/C) mutations a relative rate of 2.15, yielding $\mu_{S>W}/\mu_{S>S}=\;2.15$ (Kong et al., 2012). All mutations were treated as selectively neutral. The ancestral sequence was initialized at the expected equilibrium GC content (0.205, 0.295 nucleotide frequency for G/C and A/T respectively). We further confirmed that genomic GC% did not substantially change during the simulation by calculating nucleotide frequencies at 1) the start of the burn-in period, (2) the end of the burn-in period, and (3) the end of the simulation. At every stage, GC% was stably maintained at expected equilibrium.

The demographic model followed Gravel et al. (2011), representing three populations: ancestral African (AFR), European (EUR), and East Asian (EAS). The simulation began with an ancestral African population of N = 7,310. After a burn-in period of 10Na = 73,100 generations, the ancestral population expanded to N = 14,475. At generation 76,968, a Eurasian ancestral population (N = 1,861) split from the African population, with symmetric migration rates of 1.52×10^−4^ between the two. At generation 78,084, the European (N = 1,032) and East Asian (N = 554) populations split, with the three-way migration rates among AFR, EUR, and EAS set to 2.54×10^−5^, 7.77×10^−6^, and 3.12×10^−5^ respectively. Following the split, European and East Asian populations underwent exponential growth at rates of 0.378% and 0.478% per generation, respectively, until the final generation (79,024), at which time the simulation was halted and genomes were sampled. To mimic the number of sampled individuals in 1000 Genomes, 504 AFR, 503 EUR, and 504 EAS individuals were sampled at the final generation, and their genomes were output as VCF files. A total of 2,000 independent replicate simulations were run.

GC-biased gene conversion (gBGC) was implemented using SLiM’s gene conversion model with a gene conversion tract initiation rate of 0.7, a mean tract length of 1,500bp, and a GC transmission bias of 0.500014. This value of gBGC transmission bias parameter was determined such that the predicted equilibrium GC content under gBGC and mutation bias matches the empirical human genome-wide average of ~41% (REF). At equilibrium, the ratio of GC to AT content equals the ratio of the W>S to S>W fixation probabilities weighted by their respective mutation rates:

$$\frac{GC\%}{AT\%}=\frac{\mu_{W>S}\;\cdot \;\rho (W\;>\;S)}{\mu_{S>W}\;\cdot \;\rho (S\;>\;W)}=\frac{1}{2.15}\times \frac{\rho (W\;>\;S)}{\rho (S\;>\;W)}=\frac{0.41}{0.59}$$

which requires a fixation probability ratio of:

$$\frac{\rho (W\;>\;S)}{\rho (S\;>\;W)}=\frac{0.41\;\times \;2.15}{0.59}=1.494$$

Under gBGC, with a gBGC selection coefficient of 2b, where b is the conversion bias, the fixation probabilities of W>S and S>W mutations are given by:

$$P(W>S)=\frac{1\;-\;e^{-2N_{e}(2b)}}{1\;-\;e^{-4N_{e}(2b)}}$$

$$P(S>W)=\frac{1\;-\;e^{2N_{e}(2b)}}{1\;-\;e^{4N_{e}(2b)}}$$

If we define the population-level gBGC strength as B=4Neb, the fixation probability ratio can be reduced to:

$$\frac{\rho (W>S)}{\rho (S>W)}=\frac{1-e^{-B}}{1-e^{-2B}}\times \frac{1-e^{2B}}{1-e^{B}}=e^{B}.$$

Setting $e^{B}=1.494$ gives B=0.4015; plugging in the ancestral effective population size $N_{e}=7310$:

$$b=\frac{0.4015}{4\times 7310}\approx 1.4\times 10^{-5}.$$

The SLiM gene conversion bias parameter corresponds to 0.5+b, yielding a value of 0.500014, which was used in the mutation-bias-plus-gBGC simulation.

As a control, we ran a second set of simulations implementing mutation bias alone, without gBGC. All parameters were identical to the “mutation bias + gBGC” scenario described above, with two exceptions: the gene conversion model was omitted entirely, and the ancestral sequence was initialized at the corresponding mutation-bias-only equilibrium GC content of ~32% (i.e., 0.159, 0.341 nucleotide frequency for G/C and A/T respectively).

From each replicate’s VCF output, we extracted two summary statistics. For each polymorphic site, we computed the derived allele frequency (DAF) in each population; global DAF (across all three populations) was used to assign sites to frequency bins (DAF < 0.05; DAF > 0.95; 0.05 ≤ DAF ≤ 0.95, and all) to ensure consistent bin membership across populations. Within each frequency bin, we computed: (1) mean DAF separately for S>W and W>S mutations in each population; and (2) average GC% for each population using population specific AF values, as described earlier. 95% confidence intervals were estimated using block bootstrapping as described earlier.

## Supplementary Material

Supplement 1

Supplement 2

## Figures and Tables

**Table 1.** Average DAF for each population group in 1000 Genomes at a given mutation type along with total AT and GC% across SNPs in different variant bins stratified by DAF: all, common (5% <= DAF <= 95%), rare (DAF<5% & DAF>95%).

All SNPs, n=35,792,511									
Mutation Type		C>T	C>A	C>G	T>C	T>G	T>A	AT%	GC%
SNP Abundance		36.72%	9.86%	8.28%	29.59%	7.97%	7.58%		
		Average DAF							
Population	AFR	3.42%	2.97%	3.17%	5.74%	3.30%	3.31%	45.64%	54.36%
	EUR	3.43%	2.99%	3.16%	5.72%	3.28%	3.31%	45.65%	54.35%
	AMR	3.43%	2.98%	3.16%	5.73%	3.28%	3.31%	45.65%	54.35%
	EAS	3.42%	2.99%	3.15%	5.73%	3.28%	3.32%	45.65%	54.35%
	SAS	3.42%	2.99%	3.16%	5.73%	3.28%	3.31%	45.65%	54.35%
Common SNPs (5% <= DAF <= 95%), n=2,470,918									
Mutation Type		C>T	C>A	C>G	T>C	T>G	T>A	AT%	GC%
SNP Abundance		36.78%	9.07%	8.00%	31.32%	7.52%	7.31%		
		Average DAF							
Population	AFR	29.65%	29.95%	30.14%	32.89%	31.56%	30.58%	47.61%	52.39%
	EUR	34.49%	34.89%	34.78%	36.85%	35.72%	35.15%	48.25%	51.75%
	AMR	34.49%	34.89%	34.80%	36.89%	35.77%	35.18%	48.23%	51.77%
	EAS	34.66%	35.06%	34.89%	37.02%	35.83%	35.32%	48.27%	51.73%
	SAS	34.40%	34.82%	34.71%	36.83%	35.68%	35.06%	48.22%	51.78%
Rare SNPs (DAF<5% & DAF>95%), n=33,321,593									
Mutation Type		C>T	C>A	C>G	T>C	T>G	T>A	AT%	GC%
SNP Abundance		36.71%	9.92%	8.31%	29.46%	8.00%	7.60%		
		Average DAF							
Population	AFR	1.47%	1.14%	1.24%	3.60%	1.33%	1.37%	45.26%	54.74%
	EUR	1.12%	0.82%	0.90%	3.27%	1.02%	1.04%	45.22%	54.78%
	AMR	1.12%	0.82%	0.90%	3.27%	1.02%	1.04%	45.22%	54.78%
	EAS	1.10%	0.81%	0.89%	3.26%	1.01%	1.03%	45.22%	54.78%
	SAS	1.12%	0.83%	0.90%	3.28%	1.03%	1.05%	45.22%	54.78%

**Table 2.** Average DAF for each population group in our simulation ($\mu_{W>S}/\mu_{S>W}=2.15$ with gBGC) at S>W and W>S mutation types along with total AT and GC% across SNPs in different variant bins stratified by DAF: all, common (5% <= DAF <= 95%), and rare (DAF<5% & DAF>95%).

All SNPs, n=39,060,136					
Mutation Type		S>W	W>S	AT%	GC%
SNP Abundance		59.70%	40.30%		
Average DAF					
Population	AFR	8.80%	8.88%	41.98%	58.02%
	EUR	10.37%	10.46%	42.27%	57.73%
	EAS	10.59%	10.68%	42.31%	57.69%
Common SNPs (5% <= DAF <= 95%), n=11,201,378					
Mutation Type		S>W	W>S	AT%	GC%
SNP Abundance		59.60%	40.40%		
Average DAF					
Population	AFR	27.10%	27.26%	45.17%	54.83%
	EUR	34.86%	34.96%	46.42%	53.58%
	EAS	36.45%	36.50%	46.99%	53.01%
Rare SNPs (DAF<5% & DAF>95%), n=27,858,758					
Mutation Type		S>W	W>S	AT%	GC%
SNP Abundance		59.74%	40.26%		
Average DAF					
Population	AFR	2.11%	2.14%	40.67%	59.33%
	EUR	1.73%	1.76%	40.55%	59.45%
	EAS	1.63%	1.67%	40.56%	59.44%

## References

[1] Bergeron L. A., Besenbacher S., Bakker J., Zheng J., Li P., Pacheco G., Sinding M.-H. S., Kamilari M., Gilbert M. T. P., Schierup M. H., & Zhang G. (2021). The germline mutational process in rhesus macaque and its implications for phylogenetic dating. GigaScience, 10(5), giab029. 10.1093/gigascience/giab029 33954793 PMC8099771

[2] Bernardi G. (2000). Isochores and the evolutionary genomics of vertebrates. Gene, 241(1), 3–17. 10.1016/S0378-1119(99)00485-0 10607893

[3] Biddanda A., Rice D. P., & Novembre J. (2020). A variant-centric perspective on geographic patterns of human allele frequency variation. eLife, 9, e60107. 10.7554/eLife.60107 33350384 PMC7755386

[4] Boman J., Mugal C. F., & Backström N. (2021). The Effects of GC-Biased Gene Conversion on Patterns of Genetic Diversity among and across Butterfly Genomes. Genome Biology and Evolution, 13(5), evab064. 10.1093/gbe/evab064 33760095 PMC8175052

[5] Bukowski R., Guo X., Lu Y., Zou C., He B., Rong Z., Wang B., Xu D., Yang B., Xie C., Fan L., Gao S., Xu X., Zhang G., Li Y., Jiao Y., Doebley J. F., Ross-Ibarra J., Lorant A., … Xu Y. (2018). Construction of the third-generation Zea mays haplotype map. GigaScience, 7(4), gix134. 10.1093/gigascience/gix134 29300887 PMC5890452

[6] Byrska-Bishop M., Evani U. S., Zhao X., Basile A. O., Abel H. J., Regier A. A., Corvelo A., Clarke W. E., Musunuri R., Nagulapalli K., Fairley S., Runnels A., Winterkorn L., Lowy E., Paul Flicek, Germer S., Brand H., Hall I. M., Talkowski M. E., … Xiao C. (2022). High-coverage whole-genome sequencing of the expanded 1000 Genomes Project cohort including 602 trios. Cell, 185(18), 3426–3440.e19. 10.1016/j.cell.2022.08.004 36055201 PMC9439720

[7] Costantini M., Cammarano R., & Bernardi G. (2009). The evolution of isochore patterns in vertebrate genomes. BMC Genomics, 10(1), 146. 10.1186/1471-2164-10-146 19344507 PMC2678159

[8] Do R., Balick D., Li H., Adzhubei I., Sunyaev S., & Reich D. (2015). No evidence that selection has been less effective at removing deleterious mutations in Europeans than in Africans. Nature Genetics, 47(2), 126–131. 10.1038/ng.3186 25581429 PMC4310772

[9] Duret L., & Galtier N. (2009). Biased Gene Conversion and the Evolution of Mammalian Genomic Landscapes. Annual Review of Genomics and Human Genetics, 10(1), 285–311. 10.1146/annurev-genom-082908-150001 19630562

[10] Figuet E., Ballenghien M., Romiguier J., & Galtier N. (2015). Biased Gene Conversion and GC-Content Evolution in the Coding Sequences of Reptiles and Vertebrates. Genome Biology and Evolution, 7(1), 240–250. 10.1093/gbe/evu277 PMC4316630 25527834

[11] Fujiwara K., Kawai Y., Takada T., Shiroishi T., Saitou N., Suzuki H., & Osada N. (2022). Insights into *Mus musculus* Population Structure across Eurasia Revealed by Whole-Genome Analysis. Genome Biology and Evolution, 14(5), evac068. 10.1093/gbe/evac068 35524942 PMC9122283

[12] Gao Z., Zhang Y., Cramer N., Przeworski M., & Moorjani P. (2023). Limited role of generation time changes in driving the evolution of the mutation spectrum in humans. eLife, 12, e81188. 10.7554/eLife.81188 36779395 PMC10014080

[13] Geraldes A., Basset P., Smith K. L., & Nachman M. W. (2011). Higher differentiation among subspecies of the house mouse (Mus musculus) in genomic regions with low recombination: RECOMBINATION AND SPECIATION IN MICE. Molecular Ecology, 20(22), 4722–4736. 10.1111/j.1365-294X.2011.05285.x 22004102 PMC3204153

[14] Glémin S., Arndt P. F., Messer P. W., Petrov D., Galtier N., & Duret L. (2015). Quantification of GC-biased gene conversion in the human genome. Genome Research, 25(8), 1215–1228. 10.1101/gr.185488.114 25995268 PMC4510005

[15] Gou X., Shao Y., Wang X., Shi H., Yu J., Li X., & Guo T. (2024). Evolutionary patterns of DNA base composition at polymorphic sites highlight the role of the environment in shaping barley and rice genomes. The Plant Genome, 17(2), e20456. 10.1002/tpg2.20456 38688857 PMC12807359

[16] Gravel S., Henn B. M., Gutenkunst R. N., Indap A. R., Marth G. T., Clark A. G., Yu F., Gibbs R. A., The 1000 Genomes Project, Bustamante C. D., Altshuler D. L., Durbin R. M., Abecasis G. R., Bentley D. R., Chakravarti A., Clark A. G., Collins F. S., De La Vega F. M., Donnelly P., … McVean G. A. (2011). Demographic history and rare allele sharing among human populations. Proceedings of the National Academy of Sciences, 108(29), 11983–11988. 10.1073/pnas.1019276108 PMC3142009 21730125

[17] Haller B. C., & Messer P. W. (2023). SLiM 4: Multispecies Eco-Evolutionary Modeling. The American Naturalist, 201(5), E127–E139. 10.1086/723601 PMC10793872 37130229

[18] Harr B., Karakoc E., Neme R., Teschke M., Pfeifle C., Pezer Ž., Babiker H., Linnenbrink M., Montero I., Scavetta R., Abai M. R., Molins M. P., Schlegel M., Ulrich R. G., Altmüller J., Franitza M., Büntge A., Künzel S., & Tautz D. (2016). Genomic resources for wild populations of the house mouse, Mus musculus and its close relative Mus spretus. Scientific Data, 3(1), 160075. 10.1038/sdata.2016.75 27622383 PMC5020872

[19] Harris K. (2015). Evidence for recent, population-specific evolution of the human mutation rate. Proceedings of the National Academy of Sciences, 112(11), 3439–3444. 10.1073/pnas.1418652112 PMC4371947 25733855

[20] Harris K., & Pritchard J. K. (2017). Rapid evolution of the human mutation spectrum. eLife, 6, e24284. 10.7554/eLife.24284 28440220 PMC5435464

[21] Hershberg R., & Petrov D. A. (2010). Evidence That Mutation Is Universally Biased towards AT in Bacteria. PLoS Genetics, 6(9), e1001115. 10.1371/journal.pgen.1001115 20838599 PMC2936535

[22] Hwang D. G., & Green P. (2004). Bayesian Markov chain Monte Carlo sequence analysis reveals varying neutral substitution patterns in mammalian evolution. Proceedings of the National Academy of Sciences, 101(39), 13994–14001. 10.1073/pnas.0404142101 PMC521089 15292512

[23] Keightley P. D., & Jackson B. C. (2018). Inferring the Probability of the Derived vs. The Ancestral Allelic State at a Polymorphic Site. Genetics, 209(3), 897–906. 10.1534/genetics.118.301120 29769282 PMC6028244

[24] Keightley P. D., Trivedi U., Thomson M., Oliver F., Kumar S., & Blaxter M. L. (2009). Analysis of the genome sequences of three *Drosophila melanogaster* spontaneous mutation accumulation lines. Genome Research, 19(7), 1195–1201. 10.1101/gr.091231.109 19439516 PMC2704435

[25] Kong A., Frigge M. L., Masson G., Besenbacher S., Sulem P., Magnusson G., Gudjonsson S. A., Sigurdsson A., Jonasdottir A., Jonasdottir A., Wong W. S. W., Sigurdsson G., Walters G. B., Steinberg S., Helgason H., Thorleifsson G., Gudbjartsson D. F., Helgason A., Magnusson O. Th., … Stefansson K. (2012). Rate of de novo mutations and the importance of father’s age to disease risk. Nature, 488(7412), 471–475. 10.1038/nature11396 22914163 PMC3548427

[26] Li X., Scanlon M. J., & Yu J. (2015). Evolutionary patterns of DNA base composition and correlation to polymorphisms in DNA repair systems. Nucleic Acids Research, 43(7), 3614–3625. 10.1093/nar/gkv197 25765652 PMC4402523

[27] Long H., Sung W., Kucukyildirim S., Williams E., Miller S. F., Guo W., Patterson C., Gregory C., Strauss C., Stone C., Berne C., Kysela D., Shoemaker W. R., Muscarella M. E., Luo H., Lennon J. T., Brun Y. V., & Lynch M. (2018). Evolutionary determinants of genome-wide nucleotide composition. Nature Ecology & Evolution, 2(2), 237–240. 10.1038/s41559-017-0425-y 29292397 PMC6855595

[28] Lynch M. (2010). Rate, molecular spectrum, and consequences of human mutation. Proceedings of the National Academy of Sciences, 107(3), 961–968. 10.1073/pnas.0912629107 PMC2824313 20080596

[29] Mancera E., Bourgon R., Brozzi A., Huber W., & Steinmetz L. M. (2008). High-resolution mapping of meiotic crossovers and non-crossovers in yeast. Nature, 454(7203), 479–485. 10.1038/nature07135 18615017 PMC2780006

[30] Mathieson I., & Reich D. (2017). Differences in the rare variant spectrum among human populations. PLOS Genetics, 13(2), e1006581. 10.1371/journal.pgen.1006581 28146552 PMC5310914

[31] Muyle A., Serres-Giardi L., Ressayre A., Escobar J., & Glémin S. (2011). GC-Biased Gene Conversion and Selection Affect GC Content in the Oryza Genus (rice). Molecular Biology and Evolution, 28(9), 2695–2706. 10.1093/molbev/msr104 21504892

[32] Nabholz B., Kunstner A., Wang R., Jarvis E. D., & Ellegren H. (2011). Dynamic Evolution of Base Composition: Causes and Consequences in Avian Phylogenomics. Molecular Biology and Evolution, 28(8), 2197–2210. 10.1093/molbev/msr047 21393604 PMC3144382

[33] Näsvall K., Boman J., Talla V., & Backström N. (2023). Base Composition, Codon Usage, and Patterns of Gene Sequence Evolution in Butterflies. Genome Biology and Evolution, 15(8), evad150. 10.1093/gbe/evad150 37565492 PMC10462419

[34] Ossowski S., Schneeberger K., Lucas-Lledó J. I., Warthmann N., Clark R. M., Shaw R. G., Weigel D., & Lynch M. (2010). The Rate and Molecular Spectrum of Spontaneous Mutations in *Arabidopsis thaliana*. Science, 327(5961), 92–94. 10.1126/science.1180677 20044577 PMC3878865

[35] Petrov D. A., & Hartl D. L. (1999). Patterns of nucleotide substitution in *Drosophila* and mammalian genomes. Proceedings of the National Academy of Sciences, 96(4), 1475–1479. 10.1073/pnas.96.4.1475 PMC15487 9990048

[36] Plotkin J. B., & Kudla G. (2011). Synonymous but not the same: The causes and consequences of codon bias. Nature Reviews Genetics, 12(1), 32–42. 10.1038/nrg2899 PMC3074964 21102527

[37] Šmarda P., Bureš P., Horová L., Leitch I. J., Mucina L., Pacini E., Tichý L., Grulich V., & Rotreklová O. (2014). Ecological and evolutionary significance of genomic GC content diversity in monocots. Proceedings of the National Academy of Sciences, 111(39). 10.1073/pnas.1321152111 PMC4191780 25225383

[38] Tillo D., & Hughes T. R. (2009). G+C content dominates intrinsic nucleosome occupancy. BMC Bioinformatics, 10(1), 442. 10.1186/1471-2105-10-442 20028554 PMC2808325

[39] Tong X., Han M.-J., Lu K., Tai S., Liang S., Liu Y., Hu H., Shen J., Long A., Zhan C., Ding X., Liu S., Gao Q., Zhang B., Zhou L., Tan D., Yuan Y., Guo N., Li Y.-H., … Dai F. (2022). High-resolution silkworm pan-genome provides genetic insights into artificial selection and ecological adaptation. Nature Communications, 13(1), 5619. 10.1038/s41467-022-33366-x PMC9509368 36153338

[40] Wang J., Li X., Do Kim K., Scanlon M. J., Jackson S. A., Springer N. M., & Yu J. (2019). Genome-wide nucleotide patterns and potential mechanisms of genome divergence following domestication in maize and soybean. Genome Biology, 20(1), 74. 10.1186/s13059-019-1683-6 31018867 PMC6482504

[41] Zhao Y., Dong L., Jiang C., Wang X., Xie J., Rashid M. A. R., Liu Y., Li M., Bu Z., Wang H., Ma X., Sun S., Wang X., Bo C., Zhou T., & Kong L. (2020). Distinct nucleotide patterns among three subgenomes of bread wheat and their potential origins during domestication after allopolyploidization. BMC Biology, 18(1), 188. 10.1186/s12915-020-00917-x 33267868 PMC7713161

