# Supplementary material for: Interpreting GC content differences across populations at polymorphic sites
Source: bioRxiv. 2026 May 18:2026.05.16.725686. Preprint. [Version 1] doi: 10.64898/2026.05.16.725686 (PMC13228235; doi:10.64898/2026.05.16.725686)
Supplement: Supplement 2 [file media-2.pdf]

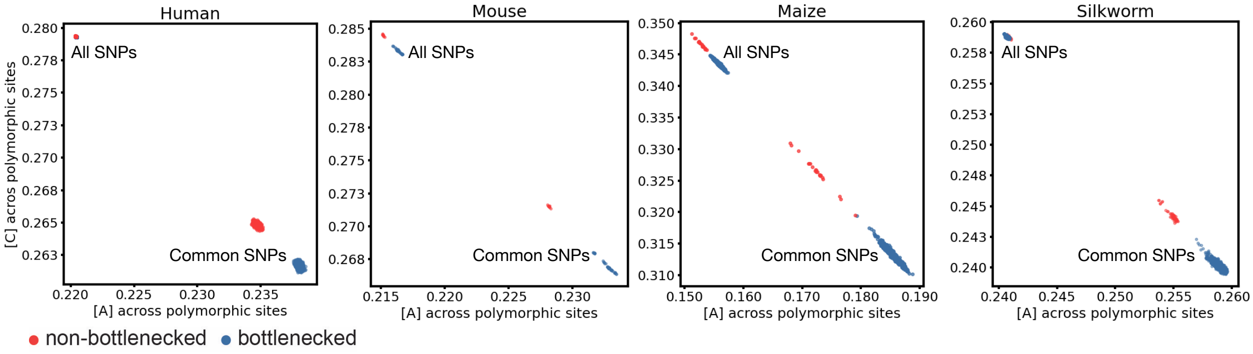

Supplementary Figure 1. Decrease in inter-population differences in GC% at SNPs is observed even after excluding singletons. Reference strand base composition plotted as proportion of A vs. proportion of C across common polymorphic sites (MAF≥5%) and all polymorphic sites (no MAF threshold, excluding singletons) between bottlenecked and non-bottlenecked populations in human, mouse, maize, and silkworm.

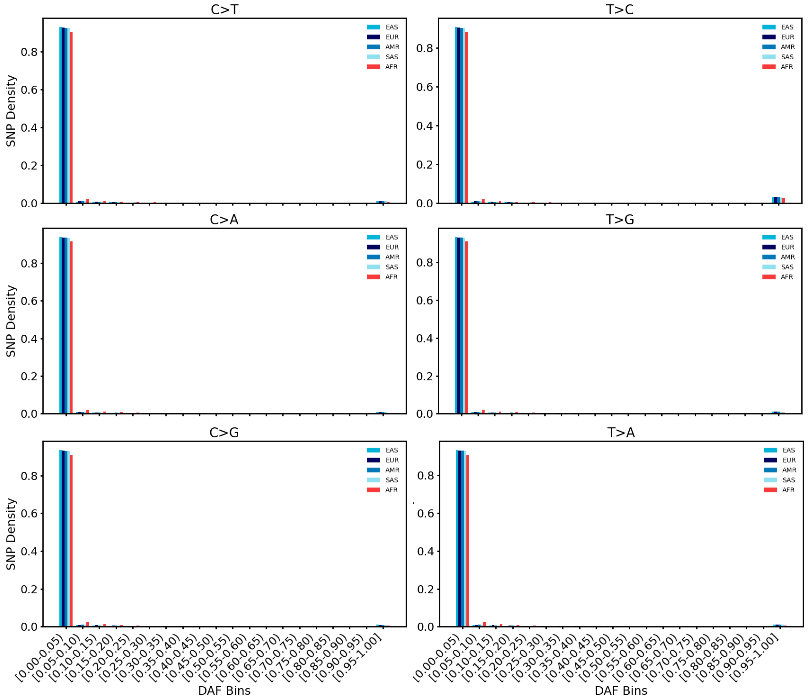

Supplementary Figure 2. Site frequency spectra (SFS) for SNPs generated by different mutation types in each population group. Full SFS for variants generated by the six mutation classes across five population groups: East Asian (EAS), European (EUR), American (AMR), South Asian (SAS), and African (AFR).

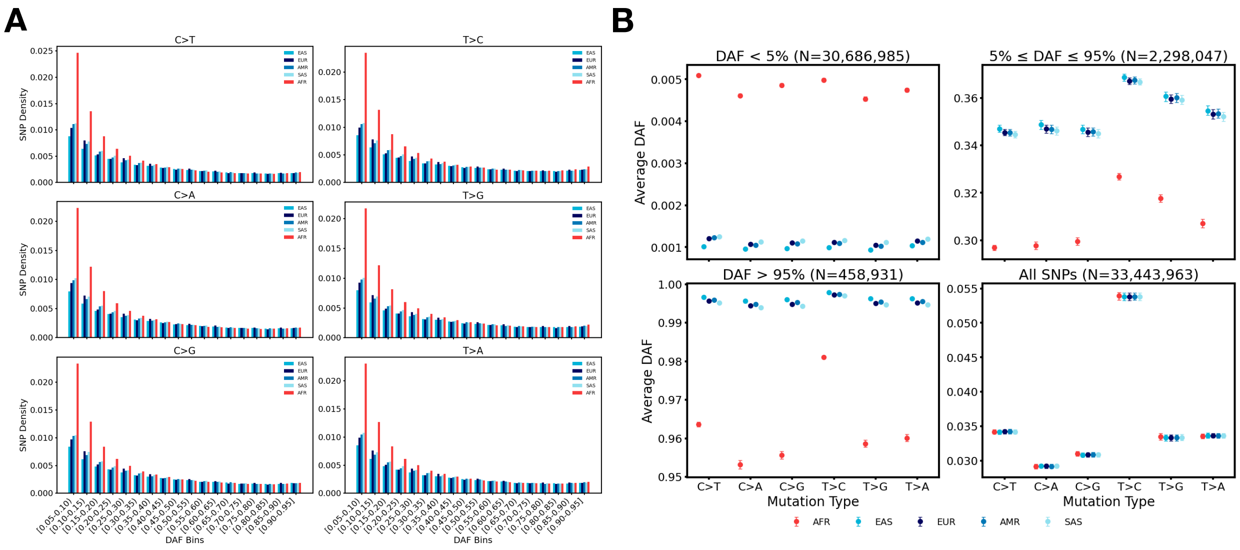

Supplementary Figure 3. Site frequency spectrum (SFS) and average derived allele frequencies (DAF) for SNPs after excluding those at CpG/TpG sites.

A. SFS for variants at non-CpG/TpG sites of each mutation type across five human population groups. Only DAF bins between 5% and 95% are shown to aid visualization. **B.** Average DAFs for each population group across different variant sets stratified by DAF after excluding CpG/TpG sites: DAF<5%, 5%≤DAF≤95%, DAF>95%, and all SNPs, for each mutation type. Error bars represent 95% confidence intervals estimated using block bootstrap (see Methods). This figure is comparable to Figure 3, but with variants at CpG/TpG sites excluded.

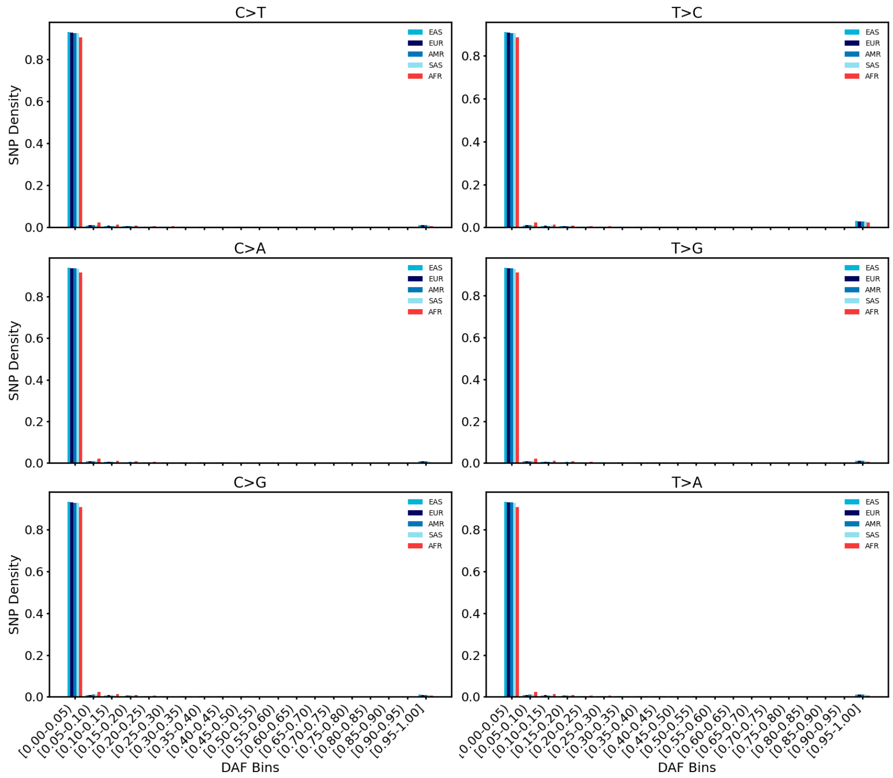

Supplementary Figure 4. Site frequency spectra (SFS) for SNPs generated by different mutation types in each population group after excluding CpG/TpG sites.
Full SFS for variants generated by the six mutation classes across five population groups: East Asian (EAS), European (EUR), American (AMR), South Asian (SAS), and African (AFR). This figure is comparable to Supplementary Figure 2, but with variants at CpG/TpG sites excluded.
